# New insight into the effects of integrated organic-inorganic fertilization on enhancing the rice straw productivity and silage quality

**DOI:** 10.5713/ab.250793

**Published:** 2026-01-05

**Authors:** Wenjie Zhang, Siran Wang, Nengxiang Xu, Chenglong Ding, Beiyi Liu

**Affiliations:** 1 Institute of Animal Science, Jiangsu Academy of Agricultural Sciences, Nanjing, China; 2 Key Laboratory of Crop and Animal Integrated Farming, Ministry of Agriculture and Rural Affairs, Nanjing, China

**Keywords:** Economic Benefit, Fertilization Strategy, Forage Yield, Rice Straw, Silage Fermentation

## Abstract

**Objective:**

The objective of this study was to determine the overall benefits of organic-inorganic integrated fertilization for the rice straw production system, including yield, forage nutritive value, silage fermentation quality, and economic returns.

**Methods:**

The field experiment used a completely randomized design with six treatments, defined by the proportion of organic manure (OM) nitrogen substituting chemical fertilizer (CF) nitrogen: T0 (0%, unfertilized control), T1 (0% OM, 100% CF), T2 (25% OM, 75% CF), T3 (50% OM, 50% CF), T4 (75% OM, 25% CF), and T5 (100% OM, 0% CF), to assess their impacts on rice straw yield, forage quality, and silage fermentation.

**Results:**

The results from the 2023–2024 trials demonstrated that T2 performed optimally. Compared to T1, T2 showed more tiller numbers (12 *vs*. 9 per plant), and greater yields of fresh straw (18,033.43–19,483.17 *vs*. 15,474.17–15,739.34 g/kg), dry matter (DM) (5,610.20–6,061.11 *vs*. 5,361.80–5,454.20 g/kg), and grain (7,419.42–7,531.34 *vs*. 7,703.96–7,841.05 g/kg DM). Nutritionally, T2 improved straw quality by elevating crude protein and water-soluble carbohydrate content while reducing fiber components. After 60 days of ensiling, treatment T2 achieved successful and desirable fermentation quality, as evidenced by a sufficiently low pH value (~3.84), a high population of lactic acid bacteria (5.6 log_10_ cfu·g^−1^ FM), moderate lactic acid production (~3.19 g/kg DM), and minimal protein degradation reflected by an acceptably low ammonia nitrogen concentration (~2.41 g/kg total nitrogen). Economically, T2 also achieved the highest net benefit (2,568 USD·ha^−1^).

**Conclusion:**

The 75% chemical plus 25% organic fertilizer regime represents a viable strategy for achieving agricultural sustainability, as it effectively supports high grain production while also improving straw yield, quality, and silage fermentation, resulting in higher economic returns.

## INTRODUCTION

Rice (*Oryza sativa* L.) is a globally vital food crop, yielding over 782 million tons annually [1]. The resultant straw constitutes a primary agricultural by-product and a key potential feedstock for ruminant animals [2]. However, the global use of rice straw as animal feed remains limited and has failed to realize its full resource potential [1]. This underutilization is associated with agricultural practices that have long been centered on maximizing grain yield through fertilizers [3]. Although these practices are effective in increasing grain yield, they often increase the cellulose and lignin content of rice straw while reducing its nutritional value, thus limiting its applicability as feed [4,5]. At the same time, the growing emphasis on sustainable agriculture has increased interest in organic cropping systems [6]. A key challenge currently facing global agriculture is how to effectively manage nutrients to ensure food safety while improving rice straw yield and feed quality.

The primary limitation of utilizing rice straw as feed is its high fiber content, which leads to poor palatability, low digestibility, and an overall feeding efficiency of only about 30% [7]. Ensiling is a widely adopted biological treatment that effectively preserves and enhances the feed value of straw [8]. However, due to the inherent deficiency of water-soluble carbohydrates (WSC) in rice straw and the low population of naturally attached lactic acid bacteria (LAB), the ensiling process often faces challenges such as a slow initiation of fermentation and unstable quality [9]. Therefore, exploring the methods to improve the nutritional characteristics of straw raw materials through agronomic measures from the field production link is of fundamental significance for improving the quality of subsequent silage and the overall utilization efficiency.

Proper fertilization practices can enhance the nutritive value of rice straw [10]. Previous studies have shown that the combined application of organic and inorganic fertilizers can promote plant growth and improve crop quality by regulating nutrient metabolism and cycling [9]. While existing research on the combined application of organic fertilizers in rice production mainly focuses on its effects on rice grain yield and quality, nutrient utilization, and soil fertility [11,12]. However, it remains unclear whether the combined application of organic and inorganic fertilizers affects straw yield, nutrient composition, and silage fermentation quality in rice cultivation. Our hypothesis is that combined application of organic fertilizers can be used to increase the yield and quality of rice straw. Therefore, this study quantifies the impact of integrated organic-inorganic fertilization on rice straw yield and feed quality, providing a theoretical basis for valorizing straw as feed and promoting organic fertilizer use.

## MATERIALS AND METHODS

### Experimental materials and procedures

The field experiment was conducted in Baima Town (119°10′51″ E, 31°36′53″ N), Lishui District, Nanjing City, Jiangsu Province, China. This site lies within the Yangtze River Delta, a major rice-producing region characterized by a subtropical monsoon climate. The regional climate features an average annual temperature of 15.3°C, annual sunshine duration of 2,146 h, annual precipitation exceeding 1,000 mm, an active accumulated temperature (≥10°C) of 4,800°C, and a frost-free period of 234 d. The test soil was yellow-brown. The content of pH value, organic carbon, total nitrogen, available phosphorus and available potassium in the field soil were 6.75, 11.51 g·kg^−1^, 0.95 g·kg^−1^, 9.45 mg·kg^−1^, and 99.51 mg·kg^−1^, respectively.

The field experiment from May 2023 to October 2024, with six treatments defined by the proportion of organic manure (OM) nitrogen substituting chemical fertilizer (CF) nitrogen: T0 (0%, unfertilized control), T1 (0% OM, 100% CF), T2 (25% OM, 75% CF), T3 (50% OM, 50% CF), T4 (75% OM, 25% CF), and T5 (100% OM, 0% CF). Each treatment was repeated three times in a 24 m^2^ (4 m×6 m) plot with a completely randomized block design. The experimental area was surrounded by a 2.0 m buffer zone, with 0.5 m wide inter-plot ridges. The ridges were covered with plastic film to prevent fertilizer cross-contamination. Each plot was designed to allow independent water irrigation and drainage.

All treatments (T1–T5) were supplied with equivalent amounts of nitrogen (N), phosphorus (P), and potassium (K). The fertilizer application rates were as follows: 225 kg·ha^−1^ of pure nitrogen (N), 150 kg·ha^−1^ of P_2_O_5_, and 150 kg·ha^−1^ of K_2_O. The organic fertilizer used was pig manure compost (containing 1.99% N, 0.80% P_2_O_5_, and 0.78% K_2_O), and provided by organic fertilizer factory of Jiangsu Academy of Agricultural Sciences. Urea (46% pure N) was used as the nitrogen fertilizer, superphosphate (12% P_2_O_5_) as the phosphorus fertilizer, and potassium chloride (60% K_2_O) as the potassium fertilizer. Organic fertilizer, phosphorus fertilizer, potassium fertilizer, and 50% of the urea were applied as basal fertilizer in a single application, while the remaining 50% of the urea was applied as a topdressing at the rice heading stage. All fields were maintained under standard local agronomic practices for irrigation, pest, and weed management.

The rice variety used was Nanjing 9108, provided by the Grain Research Institute of Jiangsu Academy of Agricultural Sciences. Rice seedlings were raised in mid-to-early May, transplanted in mid-to-early June, and harvested in mid-to-late October each year. Each plot contained 20 rows, with 20 plants per row, planted at a spacing of 20 cm×30 cm, with one plant per hill. All the management practices were consistent across treatments. Following rice harvest, ryegrass was sown in October or November every year.

### Sample collection and analysis

At rice maturity in the 2023 and 2024 seasons, the following agronomic traits were recorded: tiller number, plant height, grain yield, and straw yield. Ten random rice plants were taken from each plot to determine the number of tillers and plant height. In each plot, 10 rows were harvested, grains and straw were separated, and then the grain yield and straw yield fresh weight were measured separately. Straw samples were oven-heated at 105°C for 0.5 h to inactivate enzymes and subsequently dried at 65°C to a constant weight. The grains were dried naturally and the DM content was calculated after weighing, then the DM yield was calculated.

In the mature period of rice each year, 10 plants with consistent growth were selected from each plot, and 10 tillers were randomly taken from each plant, removed rice panicles [10]. The samples were oven-heated at 105°C for 0.5 h to inactivate enzymes and subsequently dried at 65°C to a constant weight. After drying, the samples were ground through a 60-mesh sieve for measuring the forage quality-related indicators of rice straw. WSC, starch, and non-structural carbohydrates (NSC) were measured using the anthrone-sulfuric acid colorimetric method [7]. Crude protein (CP) was analyzed by the Kjeldahl method (AOAC [13]; method 978.04) using a Kjeldahl nitrogen determination apparatus (Kjeltec 2100; Foss). Neutral detergent fiber (NDF) and acid detergent fiber (ADF) were measured using the AOAC official enzymatic-gravimetric method 937.18 [13]. The *in vitro* digestibility of dry matter (IVDMD) was measured using a two-step pepsin-cellulase method [14].

After the test plants reached maturity in 2024, 30 uniformly grown plants were selected from each plot. The panicles were removed to obtain stem material, which was then chopped into approximately 2 cm segments. Subsequently, 300 g (fresh weight) of the chopped sample was accurately weighed, placed into polyethylene bags measuring 35 cm×30 cm, and vacuum-sealed. The samples were stored anaerobically at ambient temperature for 60 d to complete silage fermentation. After the fermentation period, the silage the bags were opened, and 20.0 g of the sample was placed into a 180 mL distilled water-containing Erlenmeyer flask, sealed, and extracted at 4°C for 24 h. After filtration, the extract was used to measure fermentation quality-related indicators of silage. An additional 10.0 g sample was placed into a sterile Erlenmeyer flask, and 90 mL of sterile water was added. The flask was shaken at 120 rpm at room temperature (25°C) for 1 h, and the extract was filtered using sterile gauze. This extract was used for LAB counting. The remaining samples were dried at 65°C to a constant weight, ground, and used for nutritional quality analysis.

The DM content and DM recovery rate (DMR) were determined using the direct drying and weighing method. CP, WSC, NDF, ADF and IVDMD were measured using the methods mentioned earlier.


(1) $$\begin{array}{l} \text{DMR }(\%)=([\text{Silage weight after opening}\times \text{Silage DM}\%]/\\ \left[\text{Original material weight before silage}\times \text{Material DM}\%]\right)\\ \times 100. \end{array}$$

The filtrate was subjected to an immediate determination of pH using an electronic pH meter (S400-Basic; Mettler-Toledo). The concentrations of lactic acid (LA), acetic acid (AA), propionic acid (PA), butyric acid (BA), and isobutyric acid (IA) were quantified by high-performance liquid chromatography (HPLC) [15]. Ammonia nitrogen (NH_3_-N) was measured using the phenol-sodium hypochlorite colorimetric method [16]. The plate count method [17] was used to quantify populations of LAB, aerobic bacteria (AB), and yeast. LAB colonies were enumerated on MRS agar (Beijing Land Bridge Technology) after incubation in an anaerobic incubator (YQX-II; CIMO Medical Instrument Manufacturing) at 37°C for 2 d, under an atmosphere of N_2_:H_2_:CO_2_ (85:5:10, v/v/v). AB colonies were cultured on Nutrient Agar (Nissui Pharmaceutical) and incubated at 37°C for 1 d. Yeasts were counted on Potato Dextrose Agar (Nissui Pharmaceutical), acidified to pH 3.5 with sterile tartaric acid solution, and incubated at 37°C for 3 d. Microbial counts were expressed as colony-forming units (CFU) per gram fresh weight and converted to logarithmic values (log_10_ CFU/g).

### Production benefits

The economic inputs in this study encompassed agricultural, mechanical, and labor inputs. Agricultural inputs include rice seeds, fertilizers, pesticides, farm tools, water, etc. Mechanical inputs mainly include ploughing, seedling raising, transplanting and harvesting. Economic inputs encompassed agricultural, mechanical, and labor costs. Outputs included the value of both rice grain and straw [18].


(2) $$\begin{array}{l} \text{Total input }(\text{USD }{\text{ha}}^{-1})=\text{Agricultural input }(\text{USD }{\text{ha}}^{-1})+\\ \text{Mechanical input }(\text{USD }{\text{ha}}^{-1})+\text{Labor input }(\text{USD }{\text{ha}}^{-1}) \end{array}$$

(3) $$\begin{array}{l} \text{Gross return }(\text{USD }{\text{ha}}^{-1})=(\text{Rice production}\times \text{Price})+\\ \left(\text{Straw production}\times \text{Price}\right) \end{array}$$

(4) $$\text{Net return }(\text{USD }{\text{ha}}^{-1})=\text{Gross return}-\text{Total input}$$

### Statistical analysis

Data from this study were analyzed by one-way analysis of variance (ANOVA) using IBM-SPSS Statistics 23 software package (IBM). The following model was applied: Y*_ij_* = *μ*+D*_i_*+C*_j_*+e*_ij_*, where Y*_ij_* is the observed dependent variable, *μ* is the overall mean, D*_i_* represents the fixed effect of treatment, C*_j_* denotes the random effect of rice straw, and e*_ij_* is the residual error. The statistical differences in growth indicators, nutrient indicators, and green reservoir fermentation indicators were measured by Tukey’s multiple comparisons. The differences in economic indicators were measured by the least significant difference (LSD). Results were considered statistically significant at p<0.05. All graphs were generated using Origin 2023 (Systat Software).

## RESULTS

### Effects of combined application of organic and inorganic fertilizers on rice yield

**Figure 1:**
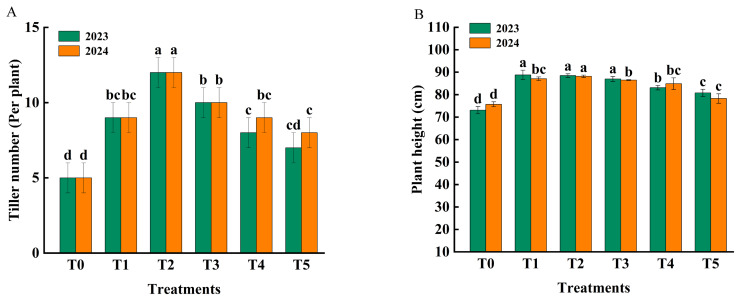
Effect of fertilization treatments (T0, T1, T2, T3, T4 *vs*. T5) on number of tillers (A) and plant height (B) of rice. T0 (no fertilizer), T1 (0% organic manure [OM], 100% chemical fertilizer [CF]), T2 (25% OM, 75% CF), T3 (50% OM, 50% CF), T4 (75% OM, 25% CF), and T5 (100% OM, 0% CF). Bars represent the treatment mean±standard error (n = 3). ^a–d^ Different lowercase letters indicate significant differences between treatments at p<0.05.

Fertilization significantly affected the number of tillers and plant height of rice (Figure 1). The tiller number in T2 was higher than in other treatments, while T1, T3 and T4 showed similar tiller numbers. In both 2023 and 2024, tiller number was greater in T2 (12 per plant) than in T1 (9 per plant). Plant height decreased with increasing organic fertilizer ratio across the two years, though T1, T2 and T3 did not differ statistically.

**Figure 2:**
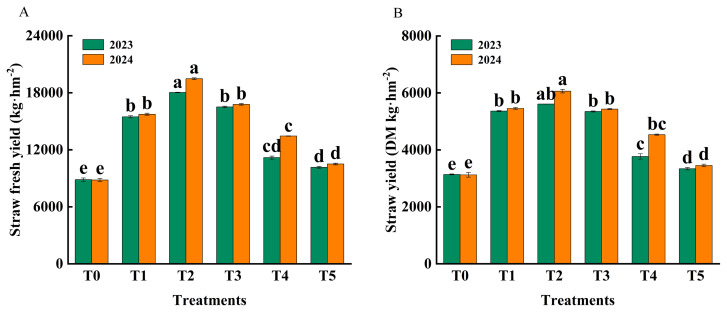
Effect of fertilization treatments (T0, T1, T2, T3, T4 *vs*. T5) on fresh grass yield (A) and dry matter (DM) yield (B) of rice straw. T0 (no fertilizer), T1 (0% organic manure [OM], 100% chemical fertilizer [CF]), T2 (25% OM, 75% CF), T3 (50% OM, 50% CF), T4 (75% OM, 25% CF), and T5 (100% OM, 0% CF). Bars represent the treatment mean±standard error (n = 3). ^a–e^ Different lowercase letters indicate significant differences between treatments at p<0.05.

**Figure 3:**
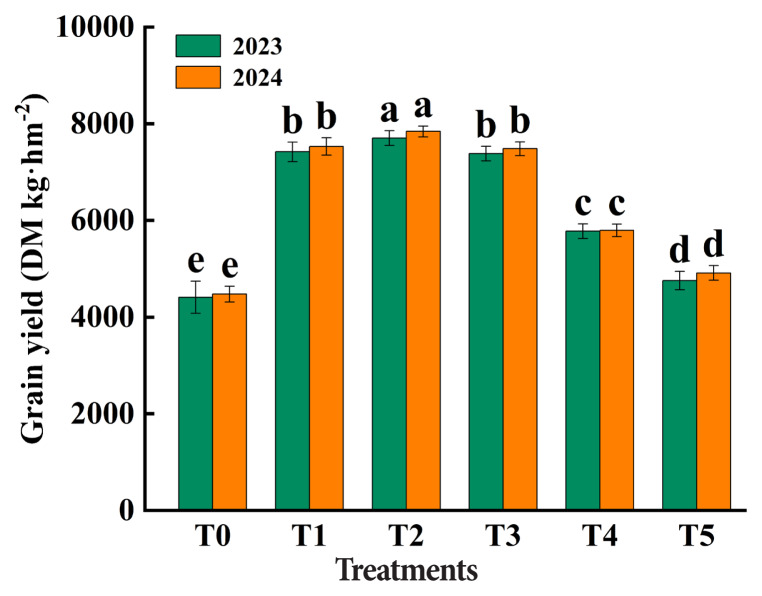
Effects of different fertilizer treatments (T0, T1, T2, T3, T4 *vs*. T5) on rice grain yield. T0 (no fertilizer), T1 (0% organic manure [OM], 100% chemical fertilizer [CF]), T2 (25% OM, 75% CF), T3 (50% OM, 50% CF), T4 (75% OM, 25% CF), and T5 (100% OM, 0% CF). Bars represent the treatment mean±standard error (n = 3). ^a–e^ Different lowercase letters indicate significant differences between treatments at p<0.05. DM, dry matter.

The fresh yield and dry matter yield of rice straw were significantly increased by fertilization, but the yield of rice straw decreased with the increase of the proportion of organic fertilizer (Figure 2). Compared with T1, T2 produced higher fresh yield (18,033.43–19,483.17 vs. 15,474.17–15,739.34 g/kg), dry matter yield (5,610.20–6,061.11 vs. 5,361.80–5,454.20 g/kg), and grain yield (7,419.42–7,531.34 vs. 7,703.96–7,841.05 g/kg DM) in 2023 and 2024. Relative to T1, T2 showed increases of 16.54% and 23.79% in fresh yield, 4.63% and 11.13% in dry matter yield, and 3.84% and 4.11% in grain yield in 2023 and 2024, respectively. Straw yield (Figure 2) and grain yield (Figure 3) were similar between T1 and T3, and both were higher than T4 and T5.

### Effects of combined application of organic and inorganic fertilizers on nutritional quality of rice straw

**Figure 4:**
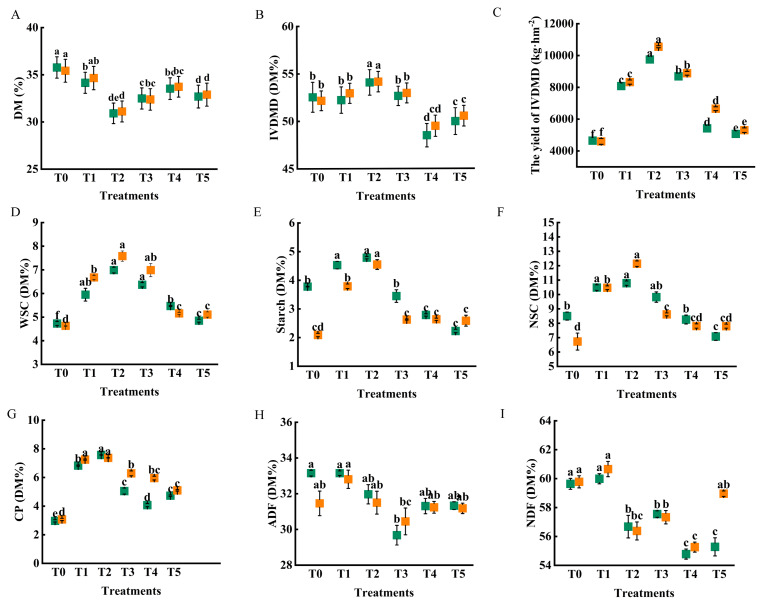
Effect of different fertilization treatments (T0, T1, T2, T3, T4 *vs*. T5) on the nutritional quality of rice straw. (A) Dry mater (DM); (B) *in vitro* digestibility of dry matter (IVDMD); (C) the yield of IVDMD; (D) water-soluble carbohydrates (WSC); (E) starch; (F) non-structural carbohydrates (NSC); (G) crude protein (CP); (H) acid detergent fiber (ADF); (I) neutral detergent fiber (NDF). T0 (no fertilizer), T1 (0% organic manure [OM], 100% chemical fertilizer [CF]), T2 (25% OM, 75% CF), T3 (50% OM, 50% CF), T4 (75% OM, 25% CF), and T5 (100% OM, 0% CF). Scatter plots represent the treatment mean±standard error (n = 3). ^a–f^ Different lowercase letters indicate significant differences between treatments at p<0.05. Green color represents the 2023 sample data, and orange color represents the 2024 sample data.

Fertilization treatments significantly affected the nutritional quality of straw (Figure 4). Over two consecutive years, data demonstrated that the combined application of organic and CFs reduced the DM content of straw. The T2 treatment exhibited the highest IVDMD (54.08% in 2023 and 54.16% in 2024), which was 20.67% and 26.64% higher than that of T1, respectively. The IVDMD values were similar among the T0, T1, and T3 treatments, and all were higher than those of T4 and T5. Fertilization treatments affected straw nutritional composition. Over two consecutive years, the WSC content consistently ranked across treatments as T2>T3>T1>T4>T5. Compared to T1, WSC content in T2 increased by 17.48% in 2023 and 13.48% in 2024. In contrast, both starch and NSC contents decreased with a higher proportion of organic fertilizer, although their levels in T2 remained above those in T1. CP content responded similarly: it increased at an appropriate organic fertilizer ratio (T2) - rising by 10.83% (2023) and 8.55% (2024) relative to T1 - but declined as the ratio continued to rise (Figure 4). Meanwhile, the contents of NDF) and ADF showed a non-linear response, initially decreasing and then increasing with more organic fertilizer. The NDF content was lower in T2, T3, and T4 than in T1, while ADF content was significantly lower only in T3.

### Effects of combined application of organic and inorganic fertilizers on the nutritional quality of straw silage

**Figure 5:**
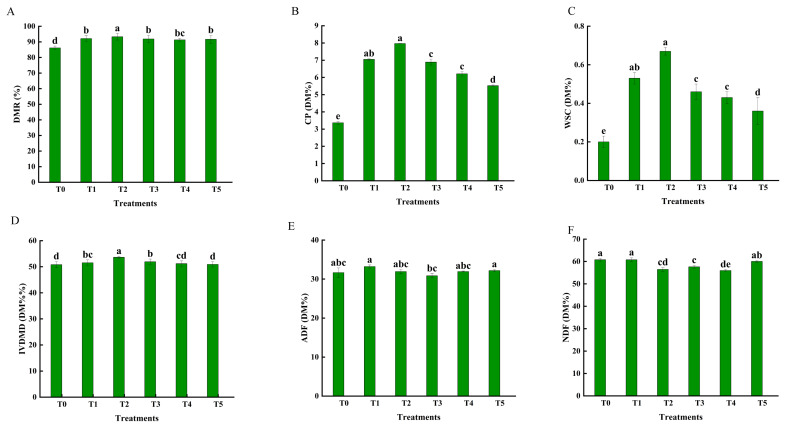
Effect of different fertilization treatments (T0, T1, T2, T3, T4 *vs*. T5) on the nutritional quality of rice straw silage. (A) Dry matter recovery rate (DMR); (B) crude protein (CP); (C) water-soluble carbohydrates (WSC); (D) *in vitro* digestibility of dry matter (IVDMD); (E) acid detergent fiber (ADF); (F) neutral detergent fiber (NDF). T0 (no fertilizer), T1 (0% organic manure [OM], 100% chemical fertilizer ([CF]), T2 (25% OM, 75% CF), T3 (50% OM, 50% CF), T4 (75% OM, 25% CF), and T5 (100% OM, 0% CF). Bars represent the treatment mean±standard error (n = 3). ^a–e^ Different lowercase letters indicate significant differences between treatments at p<0.05.

Different fertilization treatments significantly affected the nutritional quality of straw silage (Figure 5). Compared to CK, fertilization increased the silage rice straw DMR by over 91%, with T2 being higher than other treatments, while T1, T3, T4, and T5 showed similar results. The CP content was highest in T2, followed by T1 and T3. Compared to CF alone, the combined application of organic and CF reduced the NDF content. ADF content was similar among T0, T1, T2, T4, and T5. The IVDMD decreased with increasing organic fertilizer proportion. Relative to T0, IVDMD increased by 1.47% in T1, 5.57% in T2, and 2.22% in T3. T2 had higher IVDMD than T1, whereas T3 and T4 were similar and both exceeded T5.

### Effects of combined application of organic and inorganic fertilizers on the silage fermentation quality of straw

Different fertilization treatments affected the silage fermentation quality of rice straw (Table 1). Compared to T0, the fertilization treatments (T1–T5) increased the contents of LA and AA, while decreasing pH, IA, and NH_3_-N. Relative to T1, T2 further increased AA content and reduced IA and NH_3_-N.

### Effects of combined application of organic and inorganic fertilizers on the number of microorganisms in rice straw silage

Fertilization influenced the microbial populations in rice straw before and after ensiling (Table 2). Prior to ensiling, the epiphytic LAB count was low in the unfertilized control (T0, <10^5^ cfu·g^−1^ FM) and increased in all fertilized treatments (>10^5^ cfu·g^−1^ FM). This enhancing effect of fertilization on LAB counts persisted in the resulting silage. In contrast, fertilization reduced the number of AB both before and after ensiling compared to T0. The AB counts were similar among the combined organic and CF treatments. Furthermore, yeast counts were lower in the organic fertilizer treatments than in both T0 and the CF treatment (T1), both before and after ensiling.

### Production benefits

Across different fertilization treatments, the total inputs T5>T4>T3>T2>T1. Specifically, Agricultural input of T5 was the highest, followed by T4, T3, T2 and T1, Labor input increased with the increase of organic fertilizer proportion (Table 3). This discrepancy was primarily attributed to the initial stages of the planting, involving inputs such as land preparation, fertilizers, and the corresponding labor costs, which account for a considerable proportion of the eco-economic inputs. The gross return of T2 was 4,706 USD ha^−1^, which was significantly higher than other treatments.

Gross return was highest in T2 (4,706 USD ha^−1^), exceeding other treatments. Rice revenue was similar among T1, T2, and T3, and higher than in the remaining treatments. Revenue from rice straw was greatest in T2, followed by T1 and T3, with lower values in T4 and T5. Net profit was highest in T2 (2,568 USD ha^−1^). Net profits in T1 and T3 were similar and both were higher than those in T4 and T5.

## DISCUSSION

### Effects of combined application of organic and inorganic fertilizers on rice and rice straw yield

The combined application of organic and inorganic fertilizers is an important measure for reducing nitrogen fertilizer usage in paddy fields while increasing rice yield [19]. In this study, T2 treatment could increase the tiller number, the plant height, the fresh yield, DM yield of rice straw and grain yield than T1 treatment. As the ratio of organic fertilizer increased, the yield of straw and grain showed a downward trend, among which there was no difference between T3 and T1, while T4 and T5 decreased. This is because the proper application of organic fertilizer can enhance soil fertility and promote the availability and persistence of nutrients. In contrast, when applied in excess of a certain amount, the slow-release nature of organic fertilizer fails to meet the nitrogen requirements of crops during their growth period [19,20]. Considering the yield and economic benefits, organic fertilizer substitution of 25%–50% not only does not affect the yield of rice grain and straw, but also can obtain higher economic benefits. However, Wang et al [21] found that substituting 15%–30% of CF with organic fertilizer increased rice yield and nitrogen accumulation. Moreover, statistical analysis revealed that the rice straw revenue and net profit under the T2 treatment were significantly higher than those of the other treatments (p<0.05), indicating greater economic returns. Another experiment showed that the yield was increased by 37.6% when 45% CF was replaced by organic fertilizer [22]. However, Li et al [23] reported that adding organic fertilizer to CF and replacing CF with organic fertilizer increased crop yield by 8.3% and 8.7%, respectively. Although the substitution of organic fertilizer for CF has increased crop yields to varying degrees, the extent of this variation is notably large. It is closely associated with factors such as organic fertilizer sources, substitution ratios, planting regions, land use types, and climatic conditions [24].

### Effects of combined organic and inorganic fertilizer application on the nutritional quality of rice straw

The combined application of organic fertilizer reduced the DM content of straw. This is because organic fertilizers can delay the aging of plant roots and leaves, extending the duration of photosynthesis [25]. As a result, the straw remains greener at harvest, increasing the water content in the straw and reducing the DM content [26]. The CP is an important indicator for evaluating the nutritive value of forage. In this study, compared with T1 treatment, the CP content of rice straw in T2 treatment increased by 10.83% (2023) and 8.55% (2024), respectively. This is because rice mainly absorbs ammonium nitrogen, while combined application of organic and inorganic fertilizers reduces ammonia volatilization from soil [27,28]. The activities of carbon and nitrogen acquisition enzymes such as ureaseacetyl Glucosamine and cellobiosidase were increased, and soil carbon and nitrogen metabolism enhanced [29,30]. As a result, nitrogen metabolism and accumulation in plants were enhanced, which promoted plant growth and increased protein content [31]. Carbohydrates (WSC, starch, and NSC) are important indicators affecting the palatability and intake of rice straw. Herein, the T3 treatment increased the WSC content in rice straw, and the highest starch and NSC contents were observed in the T2 treatment. This is because an appropriate proportion of organic fertilizer enhances the photosynthetic characteristics of plants, increases the activity of ribulose-1,5-bisphosphate carboxylase (RuBPCase) and sucrose phosphate synthase (SPS), and thereby promotes carbon assimilation as well as the conversion of photosynthetic products into carbohydrates [9]. However, with the increase of organic fertilizer combined application ratio (T4 and T5), the content of CP, WSC, starch and NSC in rice straw were decreased. This is because as the proportion of organic fertilizer increases, the soil carbon-to-nitrogen (C/N) ratio rises in the early stage. This triggers competition for nitrogen between soil microorganisms and crops, resulting in insufficient soil nitrogen to meet the normal growth and development needs of crops, which in turn impairs the formation and accumulation of nutrients related to crop nutritional quality [32]. The NDF and ADF are important indicators for palatability and maintaining the normal fermentation function of ruminant animals’ stomachs [33]. In this study, the combined application of organic fertilizers reduced the NDF and ADF content in rice straw, delaying the senescence and lignification of rice stems and leaves. This is consistent with our previous study, which showed that organic fertilizer application reduced the ADF and NDF contents of ryegrass [34]. The digestible nutrient content in rice straw is an important indicator of nutritive value and is related to the conversion rate of livestock products. Compared with T1 treatment, the IVDMD yields were increased by 20.67% (2023) and 26.64% (2024) in T2 treatment, respectively and 7.56% (2023) and 6.65% (2024) in T3 treatment, respectively. While the IVDMD yield of T4 and T5 treatments decreased, which is related to the reduction in CP, WSC, NSC, NDF and other nutrients [34]. The improvement of nutritional quality lays a material foundation for subsequent ensilage.

### Effects of combined application of organic and inorganic fertilizers on nutritive value and fermentation quality of rice straw silage

The silage fermentation quality was affected by the epiphytic microorganisms on forage [35]. When the number of LAB attached to the straw is insufficient, LAB fail to become the dominant microbial population during ensiling. This leads to unsatisfactory silage fermentation processes, ultimately reducing both the fermentation quality and nutritive value of the silage [36]. In this study, prior to ensiling, the straw surface in the T0 treatment had a lower population of LAB but a higher population of yeast and AB. This microbial composition ultimately resulted in poorer fermentation quality and nutritive value of the silage after ensiling. The best silage quality was observed in the T2 treatment, followed by the T3 treatment. The T4 and T5 treatments showed significant declines in silage quality. This is because high-quality silage feed is not only related to the population of LAB attached to the surface of the raw material but also to the content of fermentation substrates [35]. Herein, the contents of WSC, starch, and NSC in the T2 treatment were higher than those in the other treatments, thereby providing abundant fermentation substrates for LAB. However, as the proportion of organic fertilizer increased, the sugar content showed a decreasing trend.

The NH_3_-N reflects the degradation of CP during fermentation, which is an important parameter to evaluate the ensiling process. In this study, the NH_3_-N content in rice straw silage decreased first and then increased with the increase of organic fertilizer ratio, among them the T2 had the lowest ammonia nitrogen content. This may be associated with the rapid proliferation of LAB under the T2 treatment. These LAB produced large quantities of LA, which rapidly lowered the silage pH and thereby inhibited the growth of microorganisms (e.g., *Clostridium* spp.) as well as their proteolytic activity [37]. The higher the residual WSC content in silage, the lower the DM loss during ensiling, ultimately resulting in a higher nutritie value of the silage [17]. Herein, under the T2 treatment, the WSC content of rice straw silage was the highest, the DMR was significantly higher than that in the other treatments, and the IVDMD content reached a high level, resulting in the best nutritive value of the silage among all treatments.

Under optimal T2 treatment conditions, a relatively low lactate concentration (3.19 g/kg DM) was observed, a similar phenomenon was also reported in other rice straw silage [38]. This was mainly attributed to the low buffering capacity inherent in the rice straw feed. Buffering capacity is a key physicochemical attribute that determines the resistance of silage components to pH decline, and mature rice straw has lower CP and mineral content, resulting in a much lower buffering capacity (typically<200 mEq/kg DM) than common forage [39]. Thus, in the present study, despite the limited amount of total acid produced by fermentation, it was sufficient to rapidly overcome the weak buffer resistance of the raw material and rapidly reduce the pH to 3.84. This pH level effectively inhibited microbial activity, including LAB, leading to an automatic early termination of the fermentation process, thereby limiting further accumulation of total acids. However, there may be other factors that have not been fully elucidated behind this self-terminating fermentation mode, which is a further exploration direction for follow-up research.

## CONCLUSION

This study demonstrates that 75% chemical plus 25% organic fertilizer offers a sustainable strategy for rice production, enhancing straw yield, nutritive value, and silage quality while maintaining high grain yield and economic benefits. Future work should include long-term monitoring to comprehensively assess the sustainability of organic substitution and its effects on soil fertility and microbial ecology.

## Figures and Tables

**Table 1:** The organic acid and ammonia nitrogen content of rice straw silage as affected by different fertilization treatments

Treatment	pH	LA (g·kg^−1^ DM)	AA (g·kg^−1^ DM)	PA (g·kg^−1^ DM)	BA (g·kg^−1^ DM)	IA (g·kg^−1^ DM)	NH_3_-N (g·kg^−1^ TN)
T0	4.4±0.0^a^	2.6±0.0^d^	0.7±0.0^c^	ND	ND	2.2±0.1^a^	9.0±0.6^a^
T1	3.9±0.1^b^	2.8±0.0^bc^	0.8±0.0^b^	ND	ND	1.2±0.1^c^	3.3±0.3^d^
T2	3.8±0.0^b^	3.2±0.0^a^	0.9±0.0^a^	ND	ND	1.1±0.1^d^	2.4±0.1^e^
T3	3.8±0.0^b^	2.9±0.1^b^	0.8±0.1^b^	ND	ND	1.1±0.1^cd^	3.8±0.6^cd^
T4	3.9±0.0^b^	2.7±0.1^c^	0.8±0.0^b^	ND	ND	1.2±0.1^c^	4.2±0.2^c^
T5	3.9±0.1^a^	2.8±0.0^c^	0.8±0.0^b^	ND	ND	1.3±0.1^b^	5.5±0.1^b^

T0 (no fertilizer), T1 (0% organic manure [OM], 100% chemical fertilizer ([CF]), T2 (25% OM, 75% CF), T3 (50% OM, 50% CF), T4 (75% OM, 25% CF), and T5 (100% OM, 0% CF).

Values are the means of three independent experiments±standard errors (SE).

a–e Data in the same column in lowercase letters are significantly different among treatments (p<0.05).

LA, lactic acid; DM, dry matter; AA, acetic acid; PA, propionic acid; BA, butyric acid; IA, isobutyric acid; NH_3_-N, ammonia nitrogen; TN, total nitrogen; ND, not detected.

**Table 2:** The microbial populations in rice straw silage as affected by different fertilization treatments (Log_10_ cfu·g^−1^ FM)

Treatment	LAB		AB		Yeast	

	Pre-ensiling	After ensiling	Pre-ensiling	After ensiling	Pre-ensiling	After ensiling
T0	4.5±0.1^c^	6.6±0.0^c^	6.3±0.1^a^	5.6±0.0^a^	4.8±0.1^a^	4.9±0.1^a^
T1	5.6±0.1^b^	6.7±0.0^b^	5.9±0.1^b^	4.6±0.0^b^	4.1±0.1^b^	4.3±0.0^b^
T2	5.6±0.1^b^	7.0±0.0^a^	5.8±0.1^b^	4.6±0.0^b^	3.4±0.1^d^	3.2±0.0^d^
T3	5.7±0.1^ab^	6.9±0.0^a^	5.8±0.1^b^	4.6±0.1^b^	3.6±0.1^cd^	3.4±0.1^cd^
T4	5.7±0.1^ab^	6.8±0.0^ab^	5.8±0.1^b^	4.7±0.1^b^	3.8±0.1^bc^	3.4±0.0^c^
T5	5.7±0.1^a^	6.7±0.1^b^	5.8±0.1^b^	4.8±0.0^b^	3.9±0.1^bc^	3.5±0.1^c^

T0 (no fertilizer), T1 (0% organic manure [OM], 100% chemical fertilizer [CF]), T2 (25% OM, 75% CF), T3 (50% OM, 50% CF), T4 (75% OM, 25% CF), and T5 (100% OM, 0% CF).

Values are the means of three independent experiments±standard errors (SE).

a–d Data in the same column in lowercase letters are significantly different among treatments (p<0.05).

cfu, colony forming units; FM, fresh matter; LAB, lactic acid bacteria; AB, aerobic bacteria.

**Table 3:** Comparison of economic inputs and return between different fertilization treatments (USD ha^−1^) (2024)

Treatments	Agricultural input	Mechanical input	Labor input	Total input	Rice revenue	Rice straw revenue	Gross return	Net profit
T0	679	679	1,536	1,536	2,794^c^	174^d^	2,668^d^	1,133^d^
T1	679	706	2,096	2,096	4,196^ab^	304^b^	4,500^b^	2,404^b^
T2	679	709	2,137	2,137	4,368^a^	338^a^	4,706^a^	2,568^a^
T3	679	720	2,179	2,179	4,170^a^	3,034^b^	4,473^b^	2,293^b^
T4	679	726	2,221	2,221	3,228^b^	253^c^	3,481^c^	1,260^c^
T5	679	733	2,263	2,263	2,737^bc^	192^d^	2,930^d^	667^e^

T0 (no fertilizer), T1 (0% organic manure [OM], 100% chemical fertilizer [CF]), T2 (25% OM, 75% CF), T3 (50% OM, 50% CF), T4 (75% OM, 25% CF), and T5 (100% OM, 0% CF).

Values are the means of three independent experiments±standard errors (SE).

a–e Data in the same column in lowercase letters are significantly different among treatments (p<0.05).

## Data Availability

Upon reasonable request, the datasets of this study can be available from the corresponding author.
